# Low-Cost Microalgae Cell Concentration Estimation in Hydrochemistry Applications Using Computer Vision

**DOI:** 10.3390/s25154651

**Published:** 2025-07-27

**Authors:** Julia Borisova, Ivan V. Morshchinin, Veronika I. Nazarova, Nelli Molodkina, Nikolay O. Nikitin

**Affiliations:** 1 NSS Lab, AI Institute, ITMO University, St. Petersburg 197101, Russia; 2 GreenTech, ITMO University, St. Petersburg 197101, Russia

**Keywords:** cell concentration, computer vision, microscopy, cell segmentation, microalgae

## Abstract

**Highlights:**

**What are the main findings?**

- This study presents a low-cost, automated method for estimating microalgae cell concentration using classical computer vision techniques, achieving a Pearson’s correlation coefficient of 0.96 compared to manual counts.
- The proposed approach processes images in under 30 s, offering interpretability and adaptability for laboratories with limited resources.

**What are the implications of the main findings?**

- This method bridges the gap between manual counting and expensive automated systems, making cell concentration estimation accessible for academic and research settings.
- It provides a scalable solution for hydrochemistry, biofuel production, and ecological studies, with potential applications in other microbiological fields.

**Abstract:**

Accurate and efficient estimation of microalgae cell concentration is critical for applications in hydrochemical monitoring, biofuel production, pharmaceuticals, and ecological studies. Traditional methods, such as manual counting with a hemocytometer, are time-consuming and prone to human error, while automated systems are often costly and require extensive training data. This paper presents a low-cost, automated approach for estimating cell concentration in *Chlorella vulgaris* suspensions using classical computer vision techniques. The proposed method eliminates the need for deep learning by leveraging the Hough circle transform to detect and count cells in microscope images, combined with a conversion factor to translate pixel measurements into metric units for direct concentration calculation (cells/mL). Validation against manual hemocytometer counts demonstrated strong agreement, with a Pearson correlation coefficient of 0.96 and a mean percentage difference of 17.96%. The system achieves rapid processing (under 30 s per image) and offers interpretability, allowing specialists to verify results visually. Key advantages include affordability, minimal hardware requirements, and adaptability to other microbiological applications. Limitations, such as sensitivity to cell clumping and impurities, are discussed. This work provides a practical, accessible solution for laboratories lacking expensive automated equipment, bridging the gap between manual methods and high-end technologies.

## 1. Introduction

Calculating the cell concentration of microorganisms is an important and time-consuming task. Bacteria, fungi, and algae play essential roles in many application fields. For hydrochemistry, pharmacology, bioinformatics, ecology, and bioengineering, counting these microorganisms is important for calculation of their characteristics, such as biomass concentration and biological activity [1].

*Chlorella vulgaris* is one of the most common types of microalgae living in freshwater bodies. The ubiquity of the microorganism determines the significant degree of its study and the possibility of its artificial cultivation for various purposes including agricultural feedstock [2]. For example, oil production and fatty acid composition from biomass are available [3]. Japan, as a leader in microalgae production [4], uses it for various medical treatments: skin diseases [5], haematopoiesis [6], and cancer [7]. Research has noted the possibility of using microalgae as an important source of lipids for biofuel production [8]. The global community is testing a wide range of crop management techniques to target biomass productivity, lipid, protein, carbohydrate, and pigment content [2]. To test various cultivation techniques with shifting target culture indicators, a large number of laboratory studies are required. Currently, direct cell counting methods are used to provide high-quality operational data on the state of the culture during growth.

For this purpose, traditional counting chambers are commonly used. Manual counting methods require expert knowledge and careful examination under a microscope, which is time-consuming. Additionally, the accuracy of the results heavily depends on the skill of the specialist. As a result, automated systems for cell enumeration and concentration measurement have gained popularity. However, the high cost of specialized equipment makes these methods not widespread. As alternative, many classical computer vision (CV) and deep learning (DL) approaches have been developed for cell analysis in microscope images, but they often do not directly provide the cell concentration for the sample and still require expert intervention for recalculation cell count on image to cell concentration as cells per milliliter (cells/mL). Additionally, they are still sensitive to images characteristics such as color scheme, chamber grid position, focus, etc. The principal advantage of classical computer vision approaches over deep learning methodologies is eliminating labor-intensive data annotation requirements and model retraining procedures. By leveraging structural cell morphology features, these conventional algorithms demonstrate immediate operational capability without preliminary training, thereby enabling the development of instrumentation-agnostic systems adaptable to diverse laboratory configurations. Recent machine learning approaches [9] have demonstrated low-cost algal counting using consumer hardware but still depend on annotated training data.

**We propose an approach to the automated cell count and cell concentration estimation with microscope imagery with cold start excluding model training.** Unlike many existing solutions that focus solely on creating a cell mask or counting cells within a single image, the developed system enables the direct determination of an overall sample characteristic—the cell count per milliliter of suspension—from microscope images.

Although deep learning models for segmentation require labeled data, involve training for each specific task, and are computationally intensive, they are not well suited for rapid integration into experimental workflows. To facilitate easy and low-cost implementation, we employ classical computer vision methods that focus on the structural features of cells rather than their color.

Developed microscope images processing system include

- Cell segmentation and cell enumeration on microscope images based on a lightweight CV algorithm with small inference time;
- Cell concentration calculation based on total cells number and laboratory equipment characteristics.

At the same time, existing solutions as commercial systems require prohibitively expensive equipment, and semi-automated approaches need manual grid selection and concentration calculation.

Validation of the proposed approach was performed using real wet laboratory data from *Chlorella vulgaris* microalgae cultivation in comparison to the standard manual cell concentration estimation method using a hemocytometer.

Experimental results confirmed the effectiveness of proposed approach for cell concentration automatic estimation: Pearson correlation coefficients for automatic and manual values range from 0.92 to 0.99, with a mean percentage difference of 17.96%.

Code and data for the paper experiments are available on GitHub: https://github.com/ITMO-NSS-team/microalgae_conc, accessed on 23 July 2025.

## 2. Related Works

### 2.1. Existing Instrumentality

Direct cell counting methods can be placed into three large groups: hemocytometer variations, automated cell counters, and optical methods. Popular exciting methods with short description, which can be used for *Chlorella vulgaris* cell count presented in Table 1 (1, 2).

**The hemocytometer** is a widely utilized tool for enumerating cells, playing a crucial role in disciplines such as microbiology, hematology, and biotechnology. This device facilitates the quantitative analysis of cell suspensions and is applicable to a broad spectrum of cell types, including erythrocytes [17], leukocytes [18], platelets [19], microorganisms, and cell cultures [20].

The fundamental principle of the hemocytometer is based on utilizing a microscope to visualize and count cells within a defined volume. The hemocytometer consists of two main components: the counting chamber and a cover slip, designed with an etched grid that delineates a series of squares, each with a known volume. The process begins with the preparation of a cell suspension, which may require dilution depending on the concentration of cells present. A small volume of the cell suspension is loaded into the hemocytometer, and the cover slip is placed on top, creating a fixed depth of 0.1 mm that defines the volume of the sample being analyzed.

Several types and variations of hemocytometers exist, each tailored for specific applications or designed to enhance counting accuracy. For our experimental setup, we used the Goryaev chamber because of its precise grid design and suitable volume, which help ensure accurate enumeration across various cell concentrations. Its proven reliability in hematology and microbiology makes it a good fit for small cell sizes like microalgae. Other chambers, such as the improved Neubauer chamber, feature larger squares (1 mm²) subdivided into smaller ones (0.25 mm²), which facilitate counting in both dilute and concentrated samples. The Thoma chamber, similar to the Neubauer, has a slightly different grid pattern optimized for blood cells, while the Bürker chamber is designed specifically for bacteria and small cells at higher concentrations. The Sedgewick–Rafter chamber is larger and optimized for counting plankton and microorganisms in aquatic samples. While any of these chambers could be used depending on specific needs, the Goryaev chamber was chosen in our case due to its particular features that support accurate counting of microalgae cells.

The hemocytometer presents several advantages, including its cost-effectiveness, simplicity, and versatility across a wide range of cell types, such as blood cells, bacteria, and algae. However, it also has limitations. The manual counting process can be time-consuming and susceptible to human error, particularly with high cell concentrations or uneven distributions. The accuracy of counting significantly depends on the skill and experience of the operator [21], and at very low cell concentrations, obtaining precise counts can be challenging due to difficulties in locating sufficient cells within the grid.

The widely used solutions to reduce the time required for cell enumeration are semi-automated systems based on the application of computer vision and machine learning to microscope imagery, e.g., ImageJ [11] or Cellpose [12] (and its extensions like Cellpose+ [22], Cellpose2 [13] and CellPose-SAM [14]). Their main features are summarized in Table 1(1). In this group of approaches, the user must manually select the chamber grid region and adjust segmentation parameters such as threshold levels and object size criteria. This manual setup is still time-consuming and can affect the accuracy of the results.

**The fully automated methods** described in Table 1(2) also require initial sample preparation, which involves creating a cell suspension and loading it onto a disposable slide or directly into the device’s sample holder. Once loaded, the device automatically captures images, analyzes the data, and provides results with minimal manual effort, making the process quick and user-friendly.

However, the main drawback of these systems is their extremely high cost. While medical and industrial organizations can afford such expensive equipment many academic institutions, including universities and research laboratories, often lack the necessary funding. Therefore, there is a justified and pressing need for the development of automated cell counting and cell concentration estimation systems based on classical methods, such as using a chemocytometer, which are more affordable and accessible for educational and research purposes.

Consequently, the *hemocytometer remains a fundamental tool in cell biology* for counting and quantifying cells, providing a reliable and straightforward method for cell enumeration in many laboratory settings. Due to its accessibility and widespread adoption, this work will utilize the cell counting method based on a hemocytometer.

A summary of the advantages and novelty of the proposed approach in comparison with existing solutions is presented in the Table 2.

**Practical Accessibility Scenarios**

Our method addresses critical limitations of existing systems described in Table 1 and demonstrate how our method specifically overcomes:

- **Financial barriers**: Eliminates capital equipment and recurring consumable costs.
- **Infrastructure limitations**: Functions without specialized facilities.
- **Training gaps**: Reduces expertise threshold from hours to minutes.

### 2.2. CV Automatization of Cell Enumeration

Cell enumeration using automatic algorithms can be categorized into two similar computer vision tasks: object detection and segmentation.

#### 2.2.1. Detection

Object detection is a fundamental component of automated cell counting algorithms. In its classical form, it involves determining the centers and boundaries of the targeted objects in an image using various models and algorithms [23]. Cell detection primarily provides information about the total number of cells in an image but does not specify their shape. This approach is effective for identifying object centers or specific object types. For example, cell nuclei detection as points is presented in [24]. White blood cell identification beneath other cell types has been explored in [25] for leukemia detection. Detection of cancer cells using machine learning is also a common task, with solutions based on simple models (such as support vector machines [26]), specific features extracted from microscope images [27], and variations of deep neural networks [28,29,30].

Object detection (e.g., cell detection in microscope images) is sufficient for cell counting tasks. However, for additional information—such as the size and shape of microalgae cells—segmentation or other methods are needed to accurately delineate individual cells and analyze their morphology.

#### 2.2.2. Segmentation

Segmentation tasks involve obtaining a detailed, pixel-wise mask of objects belonging to different classes. Single-cell analysis based on these masks can enhance information about colonies [31] and make automated cell counting processes fully interpretable. Computer vision techniques are widely employed to address cell segmentation problems. Existing approaches can be broadly categorized into two main types: classical CV methods based on pixel values and deep learning methods, along with their modifications.

**Classical CV methods** have been used for microbiology and medical image analysis since the last century [32]. For example, Active Contour Methods (ACM), also known as snakes, rely on local image histograms and edge information to delineate object boundaries [33]. These methods have been further refined and remain popular for various applications [34,35]. Mathematical morphology techniques, which manipulate image structures based on shape and size, are also commonly used for segmentation tasks involving blob-like objects such as blood cells [36,37]. Additionally, statistical pattern recognition approaches analyze pixel intensity distributions and spatial patterns to distinguish objects from the background [38].

While classical CV methods are often effective and computationally efficient, they can sometimes require careful parameter tuning and may be sensitive to noise or variations in illumination. They tend to work best when object boundaries are clear and well-defined, but in more complex images with overlapping objects or uneven lighting, their performance can be more challenging. Nonetheless, they remain valuable tools, especially in scenarios where simplicity, interpretability, and quick processing are important, or when the dataset is limited.

**Deep learning models** have achieved outstanding results in image segmentation tasks that are hard to deny [39]. However, DL solutions are not always suitable, as they require large amounts of annotated data. Consequently, these models cannot be easily applied to new subjects or in conditions where labeled data by specialists is scarce. Deep learning can be effectively used either by systematically collecting and annotating datasets for training or by utilizing pre-trained models on similar data.

Most architectures for segmentation tasks are based on convolutional layers. CNNs are applicable for white blood cell nucleus segmentation [40,41], and modifications of CNNs are used for plant cell segmentation [42]. Mask R-CNN is widely employed in medical applications such as nuclear cell detection and segmentation [43] and cervical cell classification [44]. Architectures like U-Net and ResNet, which incorporate skip connections, are also used for white blood cell segmentation [45,46], placenta analysis [47], and breast cancer detection [48].

Among the state-of-the-art deep learning models for cell detection is the StarDist algorithm [49]. Its core is a modified U-Net where bounding boxes for each object are refined using star-convex polygons. This enhancement allows for accurate cell boundary detection with less computational cost compared to pixel-wise mask prediction methods.

StarDist has been extensively validated on biomedical data such as histopathology cell images [50], tumor cell detection [51], and automated cell tracking [52]. Originally designed for nuclei recognition, StarDist has also been applied to chloroplast segmentation [53] and duckweed counting [54]. Its application to plant-like objects suggests that pre-trained models based on StarDist could be suitable for segmenting microalgae cells, such as *Chlorella vulgaris*, in microscope images.

The quality of cell segmentation is crucial for systems that count cells, especially as part of human-in-the-loop approaches, which increase trust and enable verification of results by specialists.

## 3. Problem Statement

**Figure 1:**
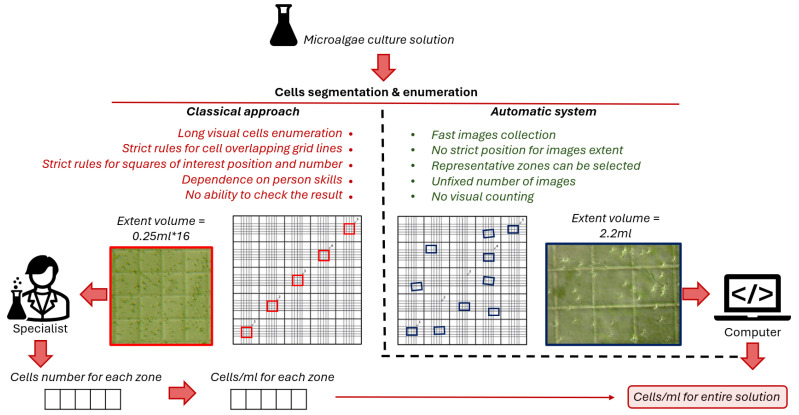
Scheme of differences between classical manual cell concentration estimation approach and proposed automatic approach.

There are variations of software for automated cell enumeration in a chemocytometer [55], but they involve manual work with images through a user interface with grid selection and subsequent manual recalculation of the resulting number of cells taking into account the chamber grid parameters (see Figure 1). Cropping images obtained from a microscope to fit the chemocytometer square requires either repetitive manual work or complex algorithms for selecting a grid on images. *To avoid image post-processing, it is necessary to calculate the volume of suspension that falls within the image coverage.*

The novelty of the proposed approach in comparison to a number of analogous methods is the automatic conversion of cell segmentation results into integral characteristic cells/mL of microalgae solution. This is achieved by converting the microscope image coverage from pixels to metric units based on the manufacturer’s stated hemocytometer grid element sizes. The recalculation procedure is performed once for the laboratory equipment used and significantly simplifies and speeds up the process of further work with it. The differences between classical manual cell counting and the proposed approach are schematically shown in the Figure 1.

## 4. Proposed Approach

The most time-consuming step in sample processing for cell count calculation is visual enumeration through a microscope for the number of squares of hemocytometer. This process can take up to 30 min per sample. In contrast, the developed system leverages computer vision, enabling the analysis of a single image at a 4k resolution in less than 30 s. We propose an automated methodology for this part of the sample processing, which reduces the total time required for concentration determination to just a few minutes per sample (including solution preparation).

*Overall, the proposed methodology consists of several key steps, which will be described in detail below:*

1. *Etalon statistics calculation (measures the edges of the chamber square in image pixels);*
2. *Laboratory equipment conversion factor calculation (calculates image volume in mL);*
3. *Run automatic cell enumeration on images and concentration calculation.*

For Steps 1 and 2, mathematical formulations were wrapped into a software module with an auto-generated calculation report and return of the image volume. The module requires a folder with cropped etalon squares and chamber characteristics. Cropping the squares from images is extremely easy for a non-expert user and takes less than a minute, but it allows parametrization of the experimental setup for a series of samples. These steps can also be performed manually if needed. Step 3 is executed programmatically using CV methods and modified mathematical formulations described below.

Cell concentration in a sample is a relative characteristic determined for a certain volume of solution. The classical approach with a hemocytometer includes the manual calculation of cells in chamber squares with a cell concentration for sample calculation using (1):

(1) $$N=\frac{a\cdot 10^{3}}{h\cdot S}=\frac{a\cdot 10^{3}}{V},$$

where *N* is the mean cells number in 1 mL of suspension (cells/mL), *a* is the mean number of cells in one square of chamber grid, *h* is the depth of chamber, *S* is the area of square, $10^{3}$ is the ${\mathrm{mm}}^{2}$ to mL conversion coefficient, and *V* is the volume of chamber square.

Each type of hemocytometer has manufacturer defined tool specification. Grid parameters and volume for most popular types of hemocytometers are presented in Table 3. Thus, when working with the (1), a known volume *V* is used under each square of the chamber and the cells are counted exclusively within the squares.

**Step 1.** In case of calculating an unknown volume of the camera that falls within the image coverage, it is necessary to use the camera grid as an etalon. The edge of the chemocytometer square with a known length in mm acts as a standard. The specialist needs to measure the edges of the square in image pixels for at least five squares located in different parts of the chamber to collect statistics. Measurements can be easily produced with built-in snipping tools of any operative system. Program module assimilate folder with snipped squares and produce statistics calculation, and future steps for image volume (2) calculation.

The average value of statistical measurements allows the formation of a *conversion factor s* from image units of measurement (pixels) to the metric system (mm): $s=\frac{l}{p}$, where *l* is specified length of chamber square and *p* is measured length of chamber in pixels.

**Step 2.** This is a necessary step for automatization is image volume calculation. This procedure should be made once for equipment setup including chamber type, microscope magnification, microscope camera resolution. Based on the conversation factor obtained in step 1 and the initial (1) the image volume is calculated using (2):

(2) $$V_{img}=\left(s\cdot P_{H}\right)\cdot \left(s\cdot P_{W}\right)\cdot h,$$

where *V* is the image volume (mL), *s* is the conversion factor from pixels to the mm, $P_{H}$ is the image height in pixels, $P_{W}$ is the image width in pixels, and *h* is the depth of the chamber.

**Step 3.** Given a volume of an area for which the number of cells present is known, the standard (1) for calculating cell concentration can be converted into a formula for calculating concentration in an automated manner (3) based on microscope images.

(3) $$N=\frac{a_{img}\cdot 10^{3}}{V_{img}},$$

where *N* is the mean cell number in 1 mL of suspension (cells/mL), $a_{img}$ is the number of cells present on the image, $10^{3}$ is the $mm^{2}$ to mL conversion coefficient, and $V_{img}$ is the image volume.

This calculation runs automatically with number of microscope images as an input for algorithm. Automation is achieved by solving the problem of detecting microalgae cells with the CV algorithm on the entire coverage of each image from the microscope.

### 4.1. Data Processing

The main disadvantage of deep learning models for segmentation task is the need for sufficient labeled data for training [60]. Preparation of such datasets is an outstanding and expensive task. Although pre-trained DL models for segmentation exist, their effectiveness on data from different subjects is often pure. That is why the basis of the proposed approach is classical computer vision algorithms, which do not involve model training.

For *Chlorella vulgaris* cell concentration estimation, the correct cell count is more important than an ideal pixel-wise mask of cell shape. This is why we reduced the task for segmentation algorithm: the system should accurately detect cells in the image by approximating them as circles. For circle finding on images, a simple and commonly used Hough transformation was used [61]. The Hough circles algorithm is implemented in the OpenCV [62] library, which is popular for circle finding on images in medicine [63], hydrochemistry [64], and robotic technology [65].

**Methodological Rationale**

The automated cell concentration estimation pipeline was designed to balance computational efficiency with biological relevance, with each processing step selected based on microalgae imaging characteristics:

1. **Spectrum correction**:
  This step enhances green-channel contrast to exploit *Chlorella vulgaris*’s chlorophyll absorption peak at 430–660 nm. Selective contrast enhancement of the green channel is performed using contrast-limited adaptive histogram equalization (CLAHE) to amplify chlorophyll-specific signals while preserving morphological details in other spectral bands. This preprocessing step improves microalgae detection robustness against illumination variation.
2. **Grayscale transformation**:
  This step reduces computational complexity while preserving morphological features. This is a luminance-preserving conversion Y=0.299R+0.587G+0.114B, where *R* is the red channel, *G* is the green channel, and *B* is the blue channel.
3. **Median blur filtering**:
  This eliminates salt-and-pepper noise from microscope optics without edge degradation. The kernel size may vary depending on the degree of image distortion and the size of the cells in the image; the default is 3. It reduces noise while preserving cell boundaries.
4. **Hough Circle detection**:
  This step leverages *Chlorella vulgaris*’s near-spherical morphology (diameter 2–10 µm). The Hough transform’s spatial constraints are defined by three interlinked geometric parameters. The radius bounds (min_radius = 15 px/3 µm and max_radius = 100 px/20 µm) establish the expected size range for *Chlorella vulgaris* cells, while dist = 100 px ensures proper separation between adjacent cells (2× maximum cell diameter). These values form a biologically grounded detection framework where
  (4) dist≥2×max_radius
  The sensitivity threshold sensitivity = 30 controls the trade-off between detection recall (lower values) and precision (higher values).
5. **Concentration calculation**:
  Converts cell counts to volumetric concentration (cells/mL) using Equation (3). The volumetric cell concentration is derived from three interdependent parameters: the raw cell count *N* obtained through automated detection, the sample-specific dilution factor D≥1, and the image volume $v_{img}$ (in mm^3^) determined by microscope chamber geometry. The relationship from Equation (3) converts 2D cell counts to 3D concentration (cells/mL), where the dilution factor *D* corrects for sample preparation protocols and $v_{img}$ is calculated from the known chamber depth and image dimensions scaled by the microscope’s pixel size. The $10^{3}$ multiplier performs unit conversion from mm^3^ to mL, with final integer rounding following standard biological reporting conventions. This formulation ensures consistency across experimental setups while maintaining physical interpretability of all parameters.

**Figure 2:**
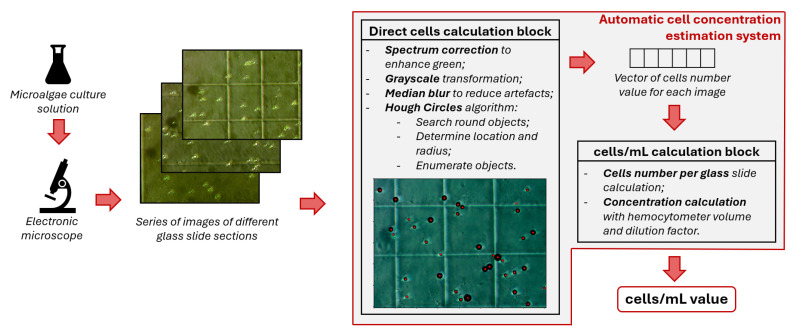
Scheme of proposed approach for cell concentration calculation.

Scheme of proposed approach is shown in the Figure 2.

### 4.2. Methodology Application Example

Our laboratory equipment includes a Goryaev chamber-type chemocytometer and a digital microscope with a magnification of 40×. Below is a modification of (1) for cell concentration estimation for our equipment that reflects the conversion of image sizes from pixels to metric units.

Microscope images have 4032 × 3024 pixels. The measurement statistics of Goryaev chamber’s grid squares (in pixels) on the validation dataset are as follows: 1222±51px (standard deviation). The Goryaev chamber manufacturer states that the length of the square side is 0.05±0.004mm; therefore, the measurement uncertainty is physically justified. So, the conversion factor $s=\frac{0.05}{1222}=4.1\cdot 10^{-5}$. Knowing the conversion factor, modifying (5) and (3), we can calculate the volume of the camera that falls within the image coverage and cell concentration:

(5) $$V_{img}=\left(s\cdot P_{H}\right)\cdot \left(s\cdot P_{W}\right)\cdot h=\left(4.1\cdot 10^{-5}\cdot 4032\right)\cdot \left(4.1\cdot 10^{-5}\cdot 3024\right)\cdot 0.1=0.002$$

(6) $$N=\frac{a_{img}\cdot 10^{3}}{V_{img}}=\frac{a_{img}\cdot 10^{3}}{0.002},$$

where *N* is the mean cells number in 1 mL of suspension (cells/mL), $a_{img}$ is the cell number detected on the microscope image, *s* is the conversion factor (mm in 1 pixel of image), $P_{H}$ is the image height in pixels (3024px), $P_{W}$ is the image width in pixels (4032px), and *h* is the depth of the chamber (0.1mm).

**Figure 3:**
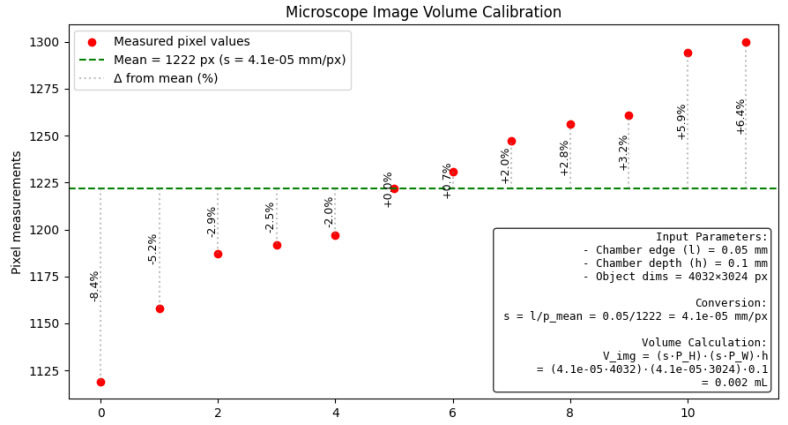
Example of auto-build report with image volume calculation for first equipment set (high-quality image—4032 × 3024).

Technical realization include an auto-build report (Figure 3) about statistic collection and image volume calculation. It use equipment parameters and a folder with cropped squares as the input and returns a visualization with calculations and the received image volume.

The advantage of this approach is the tolerance for the grid tilt and the presence of squares in the coverage of the image. Extrapolation of the concentration obtained from one image is too rough; therefore, simulating the actions of specialists, the algorithm can be applied to a photo of several different sections of the slide and averaged.

Thus, to use the modified (5), it is necessary to solve the problem of detecting microalgae cells for the entire coverage of each image from the microscope.

## 5. Validation

To evaluate the quality of the system developed in the wet laboratory, we divided the validation process into two parts:

(1) Cell detection and segmentation in microscope images;
(2) Calculation of cell concentration (cells/mL) for each microalgae culture sample.

**Figure 4:**
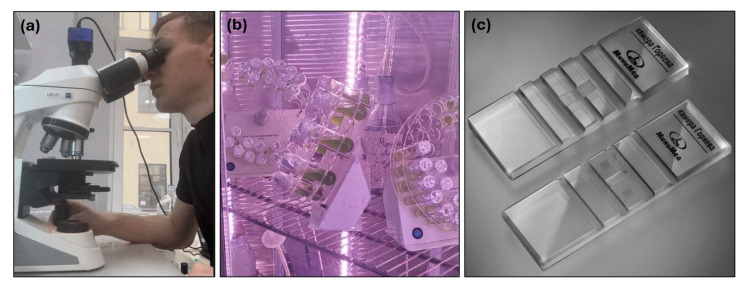
Instrumental setup for wet laboratory experiments for data collection: (**a**) digital microscope; (**b**) cultivation chamber with orbital shakers, LED illumination, and temperature regulation; (**c**) Goryaev chambers.

*Chlorella vulgaris* cell suspension was mixed on standard blue-green 11 medium (BG-11) [66] and samples for validation was selected in different cultivation days for data diversity. Our wet laboratory experimental setup presented in Figure 4.

**The validation dataset** was collected under limited data availability conditions for training deep learning models for these tasks. Two lab setups with different magnifications, mediums, and image sizes were used (Section 5.2) to demonstrate the adaptability of the approach. Samples with high cell concentrations were diluted, as the abundance of cells made manual counting difficult—specifically, the grid of the hemocytometer was not visible. The dilution method for microscope analysis aimed to ensure that each image contained approximately 10 to 60 cells. These measurements were performed to estimate the accuracy of the automatic cell concentration calculation. From existing samples, 80 randomly selected images were manually labeled to create masks for cells, serving as the ground truth for validation of detection and segmentation tasks.

### 5.1. Cell Detection and Segmentation

**Figure 5:**
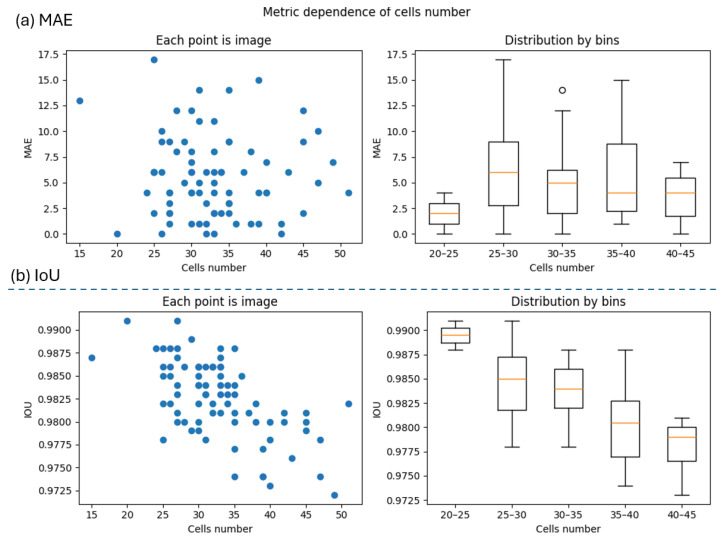
(**a**) MAE metric dependence from cells number on image—there is no significant correlation; (**b**) IoU metric dependence from cells number on image.

**Cell detection** quality was estimated by the mean absolute error (MAE)—(7). This metric is easily interpretable and makes the validation process transparent for subject specialists.

(7) $$MAE=\sum_{i=1}^{N}\left|x_{i}-y_{i}\right|,$$

where *x* is the real number of cells on image, *y* is the detected number of cells on the image, and *N* is the total number of images for validation. The MAE values for the validation dataset and their dependence on the number of cells per image are shown in Figure 5a. The MAE distribution indicates that there is no strong correlation between the error value and the number of cells in the image (with the exception of a bin with a small number of cells, which logically determines the ease of their detection). This confirms the correctness of the cell detection task and its small values indicates good quality of the CV algorithm.

**The cell segmentation** task involves creating masks that delineate individual cell objects from the initial image. To evaluate the quality of segmentation, the intersection over union (IoU or Jaccard index) metric (8) was used to compare the target masks with the automatically generated binary masks. This metric is classical for the ML segmentation task and represents the pixel-wise similarity of the real and predicted masks. The resulting masks can be utilized to extract information such as the average cell size (approximated as the radius of a circle) and the cell center position. These parameters are important for characterizing microalgae cultures.

(8) $$IoU\left(U,V\right)=\frac{\left|U\cap V\right|}{\left|U\cup V\right|},$$

where *U* represents the pixels of first class on binary mask, and *V* represents the pixels of second class on binary mask. IoU values for each image of validation set and its dependence from cells number on image presented in Figure 5b.

The distribution of IoU errors shows a decrease in the quality of predicted masks as the number of cells in the image increases. This is explained by the assumption of a round cell shape. With a larger number of objects, errors tend to accumulate, leading to reduced segmentation accuracy.

As the cold-start experimental setup provided no training data, we evaluated deep learning models pre-trained on analogous cell detection tasks. Specifically, we tested the StarDist model (pretrained on versatile fluorescent nuclei 2D_versatile_fluo) and DSB (2D_paper_dsb2018) against our microalgae microscopy images. Quality metrics for this test are presented in Table 4.

**Figure 6:**
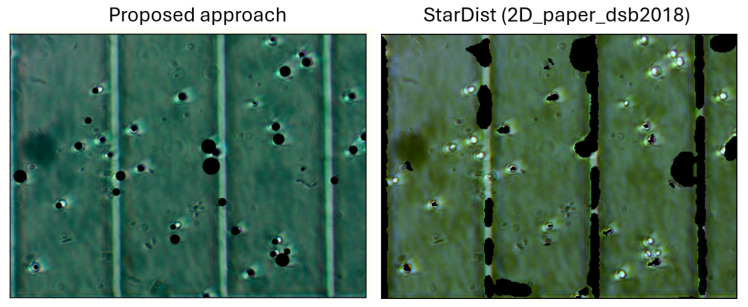
Example of StarDist pre-trained model prediction and proposed CV algorithm prediction of cells mask (black color). Color scheme changes inside model.

Comparison with pre-trained DL models confirms the necessity of training the model on microalgae task dataset to obtain satisfactory quality. When using pre-trained weights, the application of our computer vision method significantly outperforms deep learning in quality. For DL models, it is worth noting that there are a significant number of artifacts in cell segmentation associated with the presence of a chamber grid in the Figure 6. This makes the models uninterpretable and unsuitable for the task at hand.

This poor result is likely due to the model’s reliance on the brightness characteristics of the image rather than structural features. Since the grid has the same color intensity as the cells, the model tends to produce significant artifacts.

**Figure 7:**
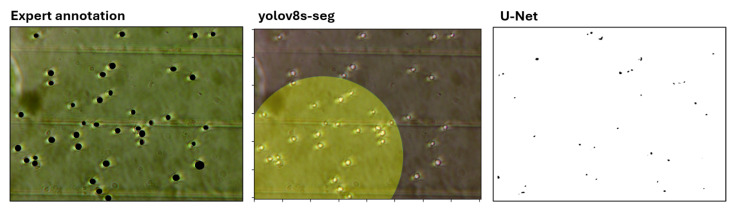
Comparison of segmentation outputs from YOLO and U-Net models trained on limited data versus ground-truth annotations. The degraded mask quality demonstrates the data requirements for deep learning approaches.

The principal advantage of our approach lies in its inherent generalizability and rapid integration into diverse experimental setups. Training task-specific deep learning models requires labor-intensive dataset labeling and becomes impractical when adapting to different laboratory equipment configurations. Nevertheless, to evaluate the feasibility of small-scale training, we conducted experiments with lightweight YOLO [67] and U-Net architectures using 60 annotated images (20 reserved for validation). Figure 7 compares the generated masks from these models against ground-truth annotations.

The segmentation results reveal two critical limitations: (1) substantial performance degradation due to insufficient training data and (2) the impracticality of manual annotation, which required approximately 2 h per equipment configuration. These findings underscore the advantage of our approach, particularly for experiments with changing imaging setups.

### 5.2. Cell Concentration Estimation

To validate the automatic cell concentration calculation algorithm, we compared its results with manual counts. Additionally, to demonstrate the approach’s applicability across different instrument configurations for monitoring *Chlorella vulgaris* cultivation, we conducted validation using two methods:

1. **High magnification and visual analysis** involves the use of samples collected during laboratory cultivation. This setup describes a real application of the proposed method in the research process: the samples have different concentrations and the environment has changed during life. For this setup, manual cell counting was performed by a laboratory technician entirely visually through a microscope; the automatic method is compared to this single expertly determined value. For this setup, a magnification of 60 was used, and the images for the automatic method were of high quality—4032 × 3024 px.
2. **Low magnification and software support** involves samples of a pure culture of *Chlorella vulgaris* with controlled dilution and additional components of the suspension. For this setup, manual cell counting was produced with the help of a graphical device for full control of the laboratory assistant working process. This setup enabled direct comparison between manual cell counting (by specialists) and automated approaches for both time requirements and concentration measurements (Section 5.2.3). By recording specialists’ cell counts for each individual chamber square, we could compare the resulting concentration distributions between manual and automated methods. Also, the use of a magnification of 40 and a lower image resolution of 2592 × 1944 px demonstrates that the approach is adaptive and can be used with different laboratory equipment.

#### 5.2.1. High Magnification and Visual Analysis

The microalgae cultivation phase was different, so we used dilution according to the direct cell counting method with a hemocytometer. The dilution coefficient accounting is included into the automatic cell/mL calculation workflow.

**Figure 8:**
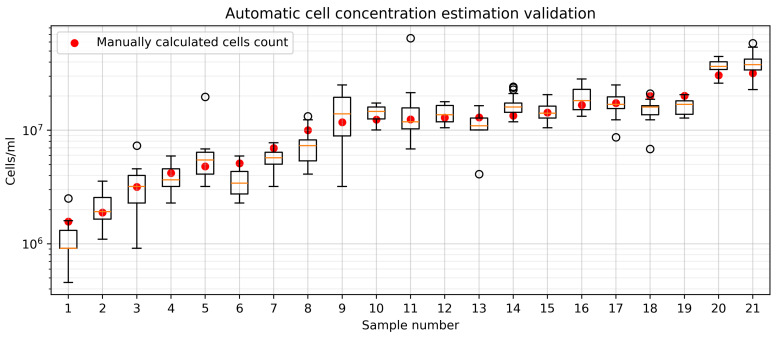
Validation of automatic cell concentration calculation system with expert values. Comparison presented with diluted suspension for clarity when comparing absolute values of different samples.

For each sample, we captured 10 microscope images from different slide zones. Applying Equation (5) to each image enabled estimation of the cell count distribution across the slide. Figure 8 compares the manually calculated cell concentrations (cells/mL) with automated results for diluted samples.

As seen in Figure 8, expert estimations lie in the intervals of the developed automatic system. This confirms the validity of the automatic system for simulating the activity of a laboratory technician when counting cells. The Pearson’s correlation coefficient for the correlation between the automatically and manually calculated values is 0.92.

Table 5 presents the cumulative cell concentration estimates (cells/mL) and associated errors for each suspension sample. For each sample, we derived the cell concentration distribution from individual microscope images, reporting both mean and median values for comparison. The percentage difference represents the absolute error between automated calculations and expert-derived values. The average percentage difference for the median is 15.24%, with a standard deviation of 10.31%. For the mean, the average percentage difference is 17.96%, with a standard deviation of 9.94%. Given these results, the use of the mean cell concentration per sample is justified, as it does not significantly differ in quality from the median. Additionally, since all parts of the chamber are technically equivalent, the choice of the mean provides a reliable estimate.

With a high correlation coefficient between manual measurements and the automated system, outliers with a large percentage difference are observed (for example, samples 11, 5, and 6). Since this setup was performed on cultured samples, some of the processed images contained clots and inclusions. The cell counting error in such images is significantly higher for both the manual and automated methods. A more detailed description of the error-producing images is described in the Section 6.

#### 5.2.2. Low Magnification and Software Support

**Figure 9:**
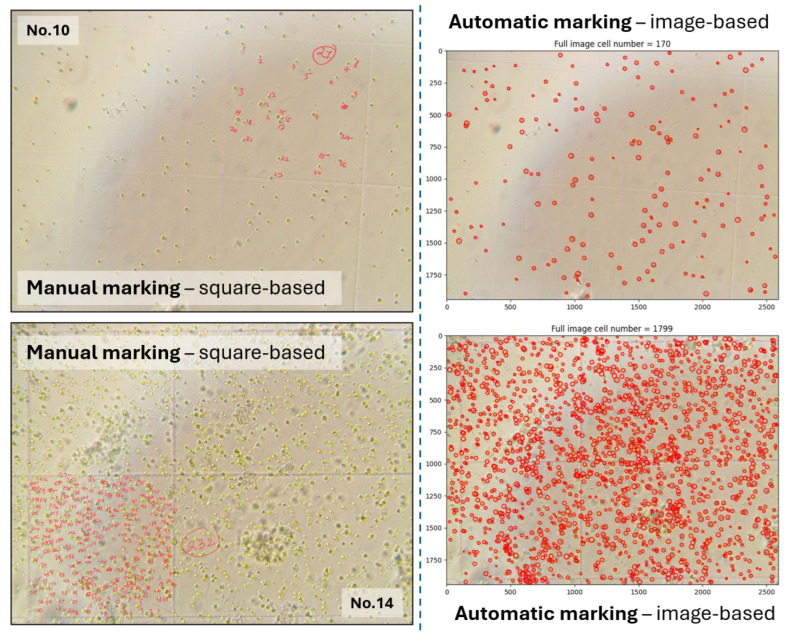
Examples of automatically generated cell markings compared with manual annotations for Samples 10 and 14, representing low and high cell concentrations, respectively.

For this experimental setup, we prepared 15 samples of the *Chlorella vulgaris* raw culture suspension with varying dilution factors and additional suspension components to ensure environmental condition variability. For validation, 10 images per sample were processed using the automated system, and 9 large squares per sample were manually annotated by an expert. Examples of manual and automated processing are shown in Figure 9.

**Figure 10:**
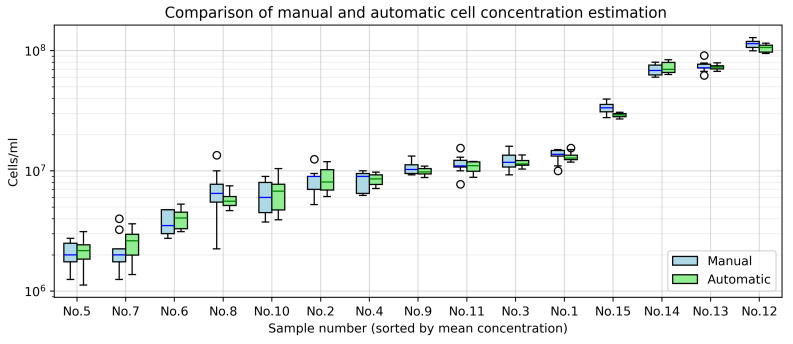
Variation in cell concentration between expert calculations (per 9 chamber squares) and the automated system (per 10 images).

The manual square-based counting approach also enables estimation of concentration distribution across the entire sample. Thus, validation results are presented as a comparison of variations between the manual and automated methods (Figure 10).

Table 6 presents validation of the automated system for cell concentration estimation against manual calculations, along with descriptions of the samples used in the low-magnification experimental setup.

**Figure 11:**
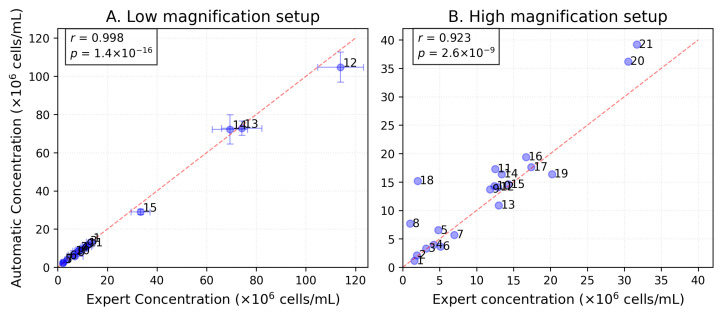
Comparison of expert and automated cell counting methods for (**A**) low-magnification (n = 15) and (**B**) high-magnification (n = 21) setups. Error bars in (**A**) represent the distribution of values among squares/images. Dashed lines indicate perfect agreement.

Statistical comparison revealed a strong agreement between the expert and automated cell counting methods (Pearson r=0.991, p<0.001), Figure 11A. Kolmogorov–Smirnov tests indicated statistically indistinguishable distributions (p>0.05) across the tested samples. The mean percentage difference across samples was 6.7±4.5%, with consistent performance across concentrations ranging from $2.0\times 10^{6}$ to $1.1\times 10^{8}\;\mathrm{cells}/\mathrm{mL}$ (Table 6).

These results demonstrate that the automated method reliably replicates expert measurements across varying cell densities. The correlation (r=0.991) and distributional similarity (p=0.855) suggest good methodological agreement. The observed relative error of (6.7±4.5)% is comparable to manual counting variability in microscopic analyses.

The largest percentage errors occurred in samples with low cell concentrations. Meanwhile, the variation in concentration values between squares or images decreased as concentration increased (Figure 10). This effect arises from the less uniform distribution of cells in samples with low concentrations.

Overall, both numerical and interval-based quality assessments confirm the effectiveness of the proposed approach in automating the cell concentration calculation process.

#### 5.2.3. Time Cost Estimation

**Figure 12:**
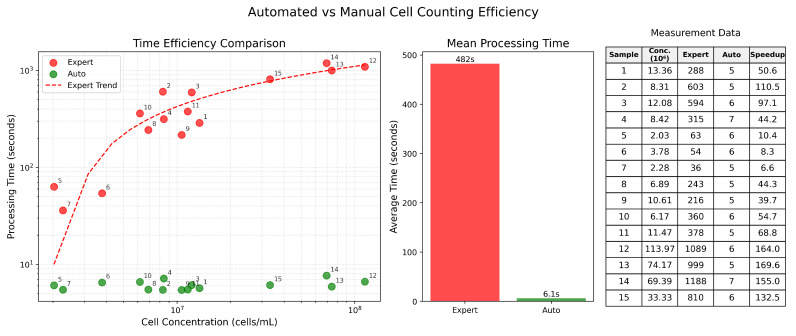
Comparison of processing times between automated and manual cell counting methods. Time measurements (in seconds) are shown for both expert and automated approaches.

The time costs for manual and automated cell counting were quantitatively evaluated during the experimental setup described in Section 5.2.2. An experienced laboratory technician annotated cells in nine large chamber squares following the standard manual concentration calculation protocol. The processing time for each sample was recorded. Notably, we excluded the time required for concentration value calculations from our measurements, as specialists typically do not perform these computations manually, and the automated calculation pipeline requires negligible time and effort. The measured processing times, including mean values and per-sample comparisons, are presented in Figure 12.

The automated system demonstrated significant time savings compared to manual processing, with increases in speed ranging from 6× for low-concentration samples to 169× for high-concentration samples. Furthermore, manual processing times exhibited a strong logarithmic increase with higher cell concentrations, as visual analysis becomes increasingly resource-intensive for human operators. In contrast, the automated system maintained consistent processing times regardless of cell concentration, showing no significant variation between low- and high-concentration samples.

#### 5.2.4. Degraded Images Processing

**Figure 13:**
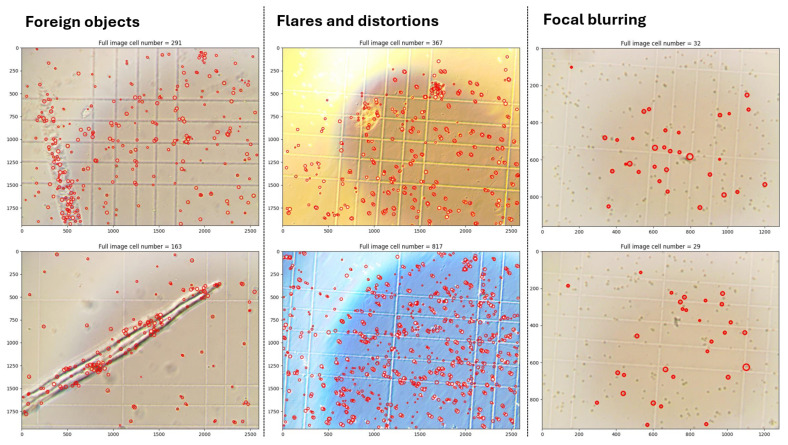
System performance on degraded images: foreign object interference, uneven illumination, and focal blurring.

To evaluate the robustness of our automated cell detection system, we analyzed its performance on intentionally degraded images. Figure 13 demonstrates the system’s outputs under suboptimal imaging conditions.

The analysis reveals three primary failure modes: (1) false positives from foreign objects coexisting with the hemocytometer grid and cells, (2) detection artifacts caused by overexposure or uneven illumination, and (3) reduced accuracy due to focal blurring. While foreign object interference requires physical sample cleanup, computational approaches could potentially mitigate illumination and focus issues. Promising solutions include

- K-means clustering for color-based image enhancement [68];
- Variational nighttime dehazing algorithms [69] adapted for microscopy.

These preprocessing techniques represent valuable directions for future system improvements, particularly for field applications where ideal imaging conditions cannot be guaranteed.

##### Experiments with Agglomeration Rate

**Figure 14:**
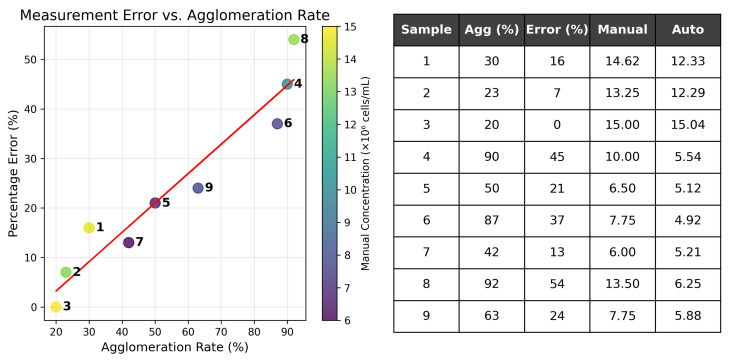
Analysis of percentage error dependency on agglomeration rate of suspension samples.

**Figure 15:**
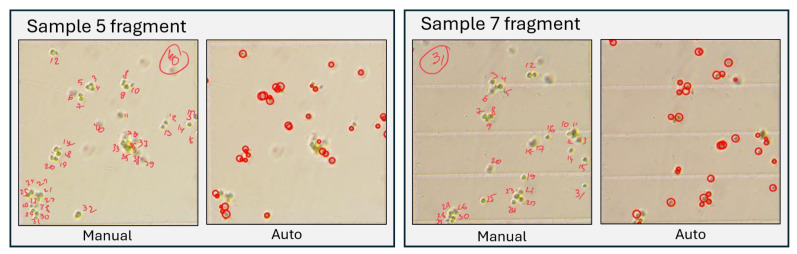
Examples of fragments of processed images with clots and their annotations.

To evaluate the proposed classical CV method’s effectiveness for cell segmentation in challenging conditions, we conducted experiments with varying agglomeration rates. These conditions were created by introducing acid injections into the suspension. An analysis of the consistency of the manual and automatic concentration measurements depending on the agglomeration rate is presented in the form of a table and a dependency plot in the Figure 14. Examples of images fragments and annotations for the manual and automatic approaches are presented on Figure 15.

Figure 14 demonstrates a strong linear dependency: the percentage error increases while the agglomeration rate increases. At the same time, the dependence is preserved for different concentrations. This fact justifies the limitation of cell counting using a chamber for both manual and automatic approaches. Non-invasive alternatives like hyperspectral imaging [70] show promise but require specialized equipment, whereas our solution uses standard microscopes.

## 6. Errors Analysis and Limitations

The system’s interpretability allows visual verification of segmentation results to identify potential counting discrepancies. These may arise either from microalgae suspension preparation issues or algorithm characteristics. Since the CV algorithm depends on structural features, microscope image color variations do not affect detection accuracy.

**Figure 16:**
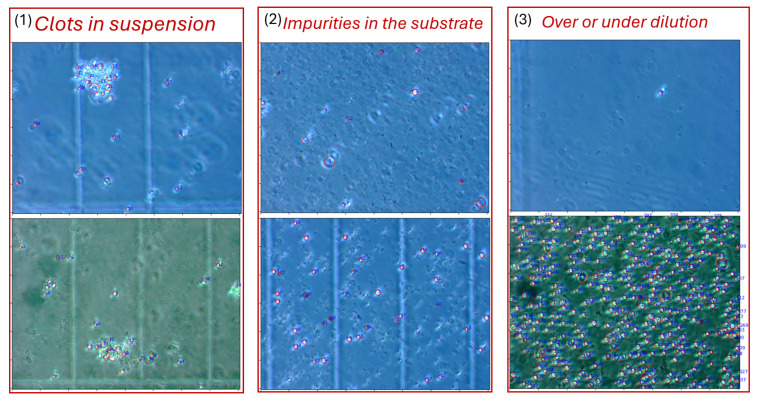
Representative examples demonstrating system limitations: (1) cell aggregates in poorly mixed suspensions (top row), (2) substrate contamination from alternative growth media (middle row), and (3) concentration artifacts from over-dilution (upper) and under-dilution (lower) samples.

The identified vulnerabilities can be grouped into three main categories. Visual examples of each are provided in Figure 16.

**1. Clots in suspension** are formed due to insufficient mixing of the sample, which causes the cells to stick together. Counting cells in such clots either manually or using computer vision methods is very difficult, so it is better to exclude them from analysis. To improve quality, the sample can be vortexed for 30 s before loading, and the chamber can be visually inspected under low magnification to verify homogeneous distribution before imaging.

**2. Impurities in the substrate.** In standard microalgae cultivation experiments, BG medium is typically used. However, when exploring alternative substrates, we conducted an experiment using a suspension of brewer’s grains. Although an expert can easily distinguish *Chlorella vulgaris* cells from other particles, the CV algorithm, which relies on structural image features, can be misled by other objects in the coverage area, resulting in incorrect detection. For reliable results, pre-filtering of samples through a 5 µm nylon mesh can be performed when testing non-standard media.

**3. Over or under dilution** of the sample can decrease the interpretability of the results. If there are no cells or only a few cells in all images of a sample, the calculation of relative concentration may be inaccurate, and additional images may be needed for clarification. Conversely, with low dilution and a high density of cells in the image coverage area, cell objects tend to overlap, making it more difficult for both the specialist to validate the results and for the algorithm to accurately detect individual cells. For correct and interpretable results, the samples can be diluted to achieve 20–50 cells per field of view, and multiple dilution series can be prepared (e.g., 1:1, 1:5, 1:10) when cell density is unknown.

**Figure 17:**
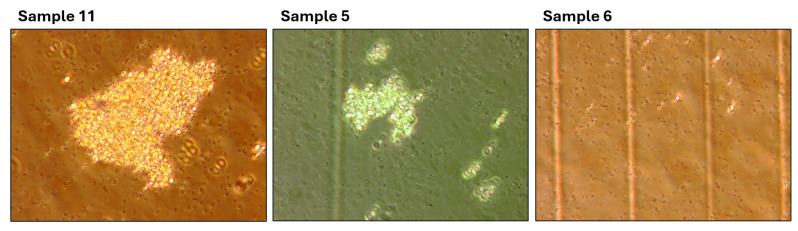
High-magnification images contributing to elevated error rates: Samples 11 and 5 exhibit cell clumping, while Sample 6 contains substrate impurities.

Analysis of high magnification setup errors (Section 5.2.1) revealed samples exhibiting the limitations described earlier. Figure 17 showcases representative images where suboptimal conditions degrade system performance.

To optimize accuracy, we recommend processing only images that meet quality standards for manual counting, as the algorithm’s performance correlates strongly with human interpretability. Both manual and automated methods are similarly affected by the described limitations.

## 7. Software Implementation

The proposed method is available as Python scripts on GitHub—https://github.com/ITMO-NSS-team/microalgae_conc, accessed on 23 July 2025. The repository contains code with the experimental setup presented in this study and guidance for users. The collected dataset, processed images, and results are also available and can be used for reproduction.

An important feature of the proposed approach is the full interpretability of the obtained results. The CV algorithm generates masks for each image a manner that imitates human activity. Examples of generated marked images are presented in Figure 9.

Providing a detailed visualization of each step of an algorithm allows for validation of the obtained results and confirms the effectiveness of human imitation, which is important for subject specialists. The concentration estimation process is transparent and trustworthy. Cumulative concentration characteristics for entire sample are reported in the console and unloaded as CSV files.

## 8. Conclusions and Discussion

The development of an automated method for microalgae cell concentration estimation represents a critical step toward improving efficiency and accessibility in hydrochemistry and related fields. Our work demonstrates that classical computer vision techniques, particularly the Hough circle transform, can provide accurate and rapid cell counting without the need for expensive equipment or extensive training data. The proposed method achieved strong agreement with manual hemocytometer counts, as evidenced by a Pearson correlation coefficient of 0.96 and a mean percentage difference of 17.96%. This performance, combined with a processing time of under 30 s per image, offers a significant improvement over traditional manual counting while maintaining interpretability through visual verification of results.

The experimental validation across two distinct setups—high magnification with visual analysis and low magnification with software support—confirmed the method’s robustness under varying conditions. In both configurations, the automated system consistently replicated expert measurements, with particularly strong agreement (Pearson r=0.991) in the controlled low-magnification environment. This adaptability suggests that the approach can be readily integrated into diverse laboratory workflows with minimal adjustments. The time efficiency gains were especially notable, with the automated system achieving increases in speed ranging from 6× to 169× compared to manual processing, depending on cell concentration.

However, several limitations warrant consideration. The accuracy of both manual and automated counts was affected by cell clumping and substrate impurities, highlighting the importance of proper sample preparation. These challenges are not unique to our method but represent fundamental constraints in microscopic cell counting. Future improvements could incorporate computational pre-processing techniques, such as variational nighttime dehazing algorithms, to enhance performance on degraded images. Additionally, while the current implementation focuses on *Chlorella vulgaris*, the underlying methodology could potentially be extended to other microbial species with similar morphological characteristics.

The practical implications of this work are particularly relevant for resource-constrained settings. By leveraging existing laboratory equipment and open-source software tools, our approach provides an accessible alternative to commercial automated cell counters, which often require substantial capital investment. The method’s interpretability, achieved through transparent intermediate processing steps and visual result verification, further enhances its value for research applications where traceability is essential.

In conclusion, this study presents a reliable and efficient computer vision-based solution of microalgae cell concentration estimation that bridges the gap between manual methods and high-end automated systems. The demonstrated combination of accuracy, speed, and accessibility addresses a critical need in both academic research and industrial applications. Future work could explore integration with user-friendly interfaces for broader adoption as well as extensions to other microbiological analysis tasks. Also, it can be used as a specialized tool for domain-specific scientific multi-agent systems based on large language models. The provided open-source implementation serves as a foundation for further development and customization by the scientific community.

## Figures and Tables

**Table 1:** Similar methods that can be used for *Chlorella vulgaris* cell count and concentration estimation.

***1. Semi-automated systems (image-based) for cell concentration estimation and cell enumeration***							
**Method**	**Essence**	**Equipment**	**Visualization Requirements**	**Time**	**Accuracy**	**Advantages**	**Disadvantages**
**Body Fluid Cell** **Counter** **“Hemo** **cytometer”** **[10]**	Manual counting with software- assisted data recording and calculations	Hemocytometer, microscope (10×–20×), PC, pipettes, stains	Clear images, 10×–20× magnification, uniform cell distribution	10–25 min/ sample	±10–20%, CV > 20% at low counts	Low cost (∼$50–100 for hemocytometer), free software, versatile, digital data storage	Labor-intensive, subjective, limited automation, low accuracy at low counts
**ImageJ** **[11]**	Semi-automated image analysis with plugins for hemocytometer or assay counts	Hemocytometer /assays, microscope with camera, PC	High-quality images, 4×–10× magnification, uniform distribution	8–18 min/ sample	<6.26% error, >97% correlation	High accuracy, fast (4.4x faster than manual), free software, flexible	Complex setup, image quality dependency, semi-automated, no viability analysis
**Cellpose** **[12,13,14]**	Semi-automated image analysis	Hemocytometer /assays, microscope with camera, PC	Low-quality images, 4×–10× magnification, uniform distribution	8–18 min/ sample	<6.26% error, >97% correlation	Fast (4.4x faster than manual), free software, flexible	Complex setup, image quality dependency, semi-automated, no viability analysis
***2. Automated systems (devices) for cell concentration estimation and cell enumeration***							
**GloCyte** **[15]**	Semi-automated fluorescence microscopy for CSF	GloCyte system, cartridges, reagents	Not applicable (automated imaging), 30 µL sample	5–8 min/ sample	Detects 1 cell/µL, CV < 20%, >97% correlation	High accuracy at low counts, fast, low sample volume, safe	High cost (∼$10,000 –20,000), CSF-specific, no differential counts, reagent dependency
**Countess** **[16]**	Automated brightfield/ fluorescence imaging	Countess device, disposable slides, stains	Not applicable, 10–50 µL sample	1–3 min/ sample	CV <5%, >95% correlation	Very fast (<30 s), accurate, viability analysis, user-friendly	High cost (∼$5000 –15,000), consumable dependency, less reliable at low counts
**ADAM** **CellT [16]**	Automated fluorescence microscopy, cGMP-compliant	ADAM CellT device, AccuChip slides, PI stains	Not applicable, 13 µL sample	2–3 min/ sample	CV <5%, >95% correlation	High accuracy, fast, regulatory compliance, viability analysis	High cost (∼$10,000 –20,000), consumable dependency, limited range
**Countstar** **[16]**	Automated brightfield/ fluorescence with AI	Countstar device, slides, stains	Not applicable, 10–50 µL sample	1–3 min/ sample	CV <5%, >95% correlation	Fast, accurate, multifunctional, versatile, data-rich	High cost (∼$10,000 –25,000), complex setup, consumable dependency

**Table 2:** Key advantages of proposed approach versus existing methods.

Feature	Commercial Systems	Semi-Automated (ImageJ/Cellpose)	Our Method
No grid selection needed	×	×	✓
Illumination robust	×	×	✓
Direct cells/mL output	✓	×	✓
Equipment cost	$5k–$25k	$0–$500	$0 *
No training data required	×	×	✓

* Uses existing lab equipment.

**Table 3:** Characteristics of different types of popular hemocytometers.

Hemocytometer Type	Grid Size (Large Square)	Number of Large Squares	Subdivisions (Small Squares)	Size of Small Square	Depth	Volume per Large Square	Typical Use
Neubauer Improved [56]	1 mm × 1 mm	3 main squares	16 per main square	0.0625 mm^2^	0.1 mm	0.1 mL	Blood cell counting
Thoma [57]	1 mm × 1 mm	1 or 4 (depending on model)	Varies	0.0625 mm^2^	0.1 mm	0.1 mL	Cell cultures, yeast, bacteria
Petroff–Hausser [58]	1 mm × 1 mm	1 (single large square)	Subdivided into smaller squares	0.0625 mm^2^ (or as specified)	0.02 mm	0.02 mL	Bacterial counting
Goryaev [59]	1 mm × 1 mm	1 (main square)	25 smaller squares (each 0.2 mm × 0.2 mm)	0.04 mm^2^	0.02 mm	0.02 mL	Sperm and small cell counting

**Table 4:** Quality metrics of different models for microalgae cell segmentation with standard deviation intervals on the validation set.

Model	MAE	IoU	Cell Area Error, %
**Proposed approach**	6±6	0.97±0.02	0.008±0.01
StarDist (2D_versatile_fluo)	44±19	0.92±0.05	3.110±5.08
StarDist (2D_paper_dsb2018)	25±23	0.93±0.04	2.411±2.88

**Table 5:** Validation of automatic method of cell concentration counting.

Sample Number	Dilution	Expert cells/mL ($10^{6}$)	Automatic cells/mL ($10^{6}$) (Median)	Percentage Difference (Median), %	Automatic cells/mL ($10^{6}$) (Mean)	Percentage Difference (Mean), %
**1**	2	1.57	0.91	**41.8**	1.14	**27.2**
**2**	5	1.89	1.92	**1.6**	2.13	**13**
**3**	1	3.17	3.2	**0.9**	3.29	**3.87**
**4**	1	4.19	3.65	**12.9**	3.95	**5.94**
**5**	1	4.81	5.48	**14.1**	6.53	**35.9**
**6**	1	5.11	3.43	**33**	3.65	**28.5**
**7**	1	6.97	5.71	**18.1**	5.71	**18.1**
**8**	1	1.00	7.31	**26.9**	7.67	**23.3**
**9**	1	11.8	13.9	**18.3**	13.7	**16**
**10**	1	12.4	14.6	**17.7**	14.3	**15.1**
**11**	1	12.5	11.9	**4.8**	17.3	**38.5**
**12**	2	12.9	13.7	**6.1**	14.1	**8.93**
**13**	1	13	11	**15.7**	10.9	**16.4**
**14**	10	13.4	16	**18.9**	16.4	**22.1**
**15**	1	14.3	14.2	**1.2**	14.6	**1.67**
**16**	1	16.7	18.3	**9.6**	19.4	**16.2**
**17**	10	17.4	16.9	**2.8**	17.6	**1.1**
**18**	2	2.00	1.6	**20.1**	15.2	**24.2**
**19**	1	20.2	16.9	**16.2**	16.4	**18.9**
**20**	1	30.5	36.5	**19.9**	36.2	**18.8**
**21**	1	31.7	37.9	**19.5**	39.2	**23.6**

**Table 6:** Validation of the automated cell concentration counting method for the low-magnification setup.

Sample	Medium (Dilution /Components)	Expert cells/mL ($10^{6}$) (Mean)	Automatic cells/mL ($10^{6}$) (Mean)	Percentage Difference (Mean), %
**1**	1	13.36	13.17	**1.5**
**2**	1	8.31	8.58	**3.2**
**3**	1.33	12.08	11.70	**3.1**
**4**	2	8.42	8.49	**0.8**
**5**	2.86	2.03	2.15	**6.2**
**6**	4	3.78	4.00	**5.9**
**7**	6.67	2.28	2.57	**12.7**
**8**	2/acid 0.1 mL	6.89	5.69	**17.4**
**9**	2/alkali 0.1 mL	10.61	9.92	**6.5**
**10**	2/NaCl 0.1 mL	6.17	6.55	**6.2**
**11**	1.33/centrifugation	11.47	10.83	**5.6**
**12**	2/centrifugation	113.97	104.77	**8.1**
**13**	2.86/centrifugation	74.17	72.82	**1.8**
**14**	4/centrifugation	69.39	72.26	**4.1**
**15**	6.67/centrifugation	33.33	29.00	**13.0**

## Data Availability

Code of the implemented approach is available in https://github.com/ITMO-NSS-team/microalgae_conc, accessed on 23 July 2025.
